# Non-anatomical revascularization of the portal vein in children with non-cirrhotic extrahepatic portal vein obstruction

**DOI:** 10.1007/s00247-025-06218-1

**Published:** 2025-03-19

**Authors:** Kessy Djonis Martins de Mattos, Paolo Marra, Francesco Saverio Carbone, Riccardo Muglia, Ludovico Dulcetta, Stefano Fagiuoli, Lorenzo D’Antiga, Sandro Sironi

**Affiliations:** 1 School of Medicine and Surgery, University of Milano-Bicocca, Piazza dell’Ateneo Nuovo 1, 20126 Milan, Italy; 2 Department of Radiology, ASST Papa Giovanni XXIII Hospital, Piazza OMS 1, 24127 Bergamo, Italy; 3 Gastroenterology Hepatology and Transplantation Unit, Department of Medicine, ASST Papa Giovanni XXIII Hospital, Bergamo, Italy; 4 Paediatric Hepatology Gastroenterology and Transplantation, ASST Papa Giovanni XXIII Hospital, Bergamo, Italy

**Keywords:** Angioplasty, Cavernous transformation, Endovascular procedures, Portal hypertension, Portal vein

## Abstract

Portal hypertension resulting from non-cirrhotic extrahepatic portal vein obstruction is a cause of severe morbidity related to variceal bleeding in children. In patients not eligible for Meso-Rex bypass, rescue interventions for refractory variceal bleeding have traditionally included surgical porto-systemic shunts or transjugular intrahepatic porto-systemic shunt (TIPS), which alter the normal physiology of liver perfusion and expose children to long-term complications. Portal vein recanalization is a recently described technique which aims to restore the hepatopetal flow through the native portal system but its feasibility is often limited. We aim to present two innovative techniques of percutaneous non-anatomical portal vein recanalization for treating non-cirrhotic extrahepatic portal vein obstruction in children who failed anatomical revascularization.

**Graphical abstract:**

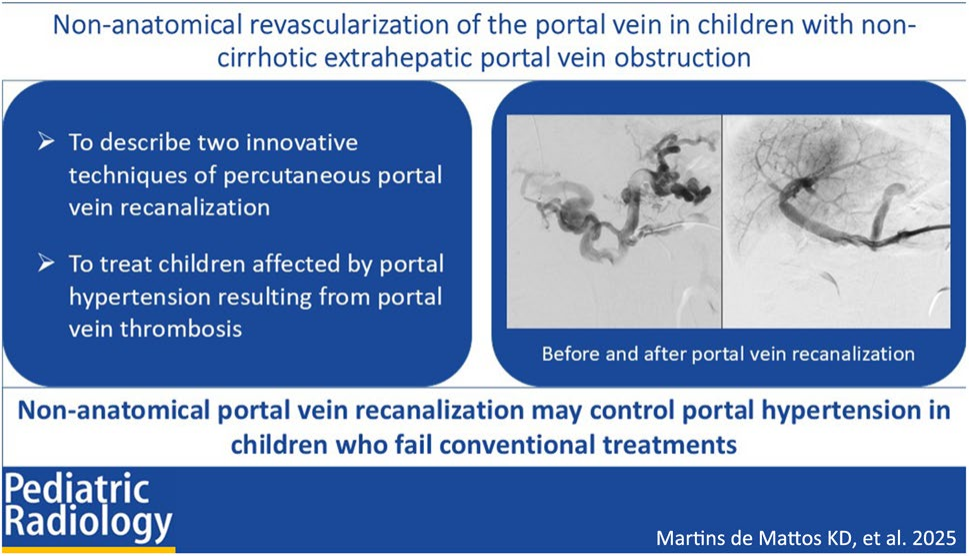

## General background

Transjugular intrahepatic porto-systemic shunt (TIPS) has been considered the treatment of choice for complications of portal hypertension in children not eligible for Meso-Rex bypass surgery [1–3]. However, distinguishing between cirrhotic and non-cirrhotic portal hypertension is crucial, as the latter may be resolved by portal vein recanalization without TIPS creation. Unlike TIPS and similar to the surgical Meso-Rex bypass, portal vein recanalization alone may restore the physiologic flow to the liver, avoiding porto-systemic shunting. Therefore, portal vein recanalization may represent an alternative to the Meso-Rex bypass and a better option compared to surgical porto-systemic shunts and TIPS [4]. Technical challenges of portal vein recanalization and a relatively low technical success rate are the main drawbacks, which make portal vein recanalization suitable only in very expert centres.

## Body of report

We have recently described the technique of portal vein recanalization in paediatric patients suffering from non-cirrhotic extrahepatic portal vein obstruction acquired in the perinatal age [5]. The reported technical success rate was 45% and recanalization was performed through the native portal tract in all the successful cases. Since that preliminary experience, we have started adopting new techniques, which resulted in an improvement of the overall technical success rate, with secondary success in two patients who had previously failed. The first technique involves the creation of a porto-portal bypass by means of the “gun-sight” technique; the second one involves exploitation of a spontaneous porto-portal collateral with angioplasty and stenting. Both the techniques consist in bypassing the native thrombosed portal tract whose catheterization failed. The study is compliant with the Declaration of Helsinki and informed consent was obtained from patients’ parents for publishing the case report.

## Technique 1

**Fig. 1:**
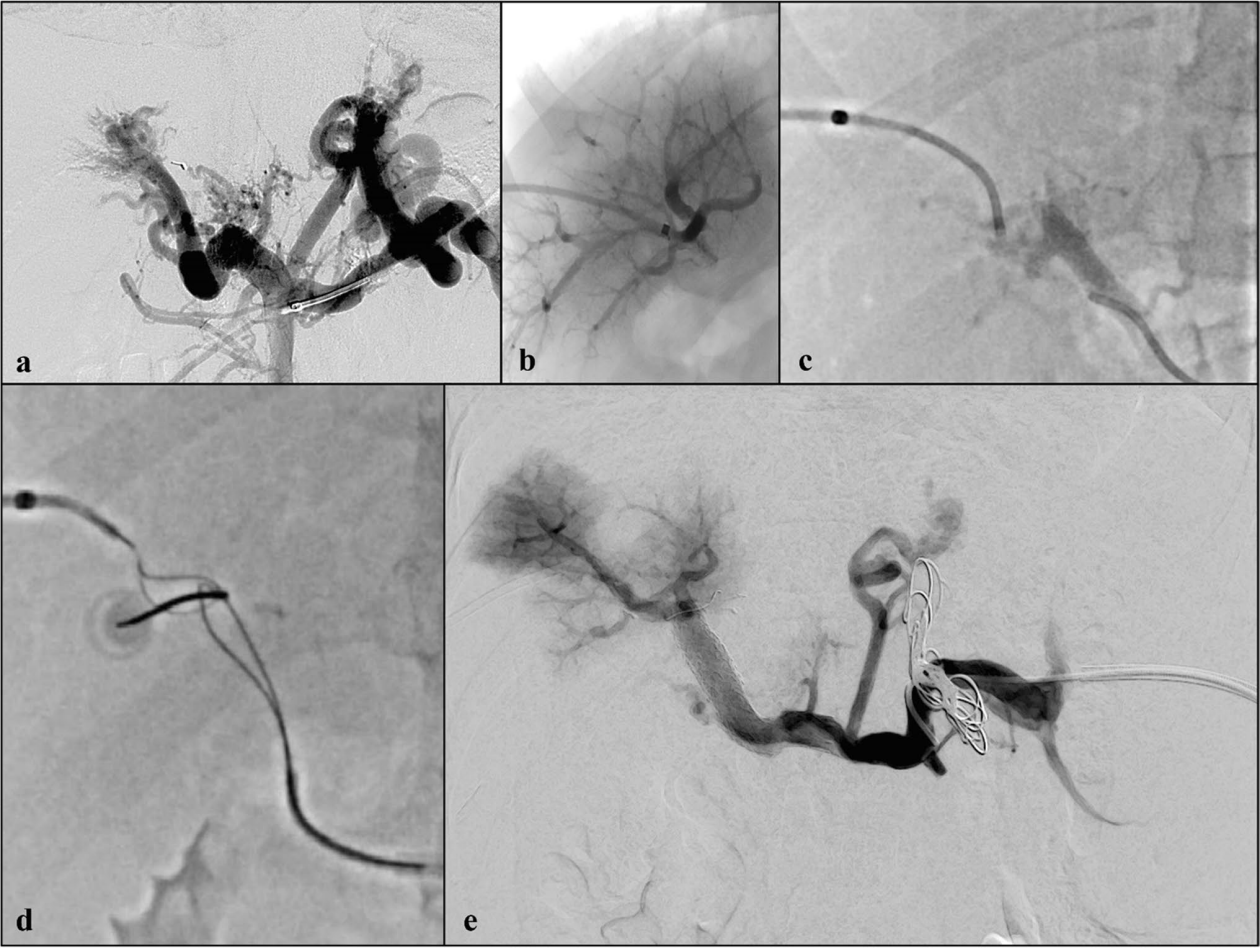
An 8-year-old boy with extrahepatic portal vein obstruction resulting from perinatal thrombosis. **a** Posteroanterior view of the transplenic digital subtraction venography shows cavernous transformation of the portal vein. **b** Right anterior oblique projection fluoroscopy image of the transhepatic venography shows a patent intrahepatic portal system. **c** Posteroanterior fluoroscopy view of simultaneous transhepatic and transplenic catheterization shows failure of anatomical recanalization with a short distance between the catheters’ tips. **d** Right anterior oblique projection fluoroscopy image shows the “gun-sight” technique with two snare catheters respectively placed from the transhepatic and transplenic accesses and coaxially targeted with a 22-G Chiba needle under fluoroscopic guidance. **e** Posteroanterior view of the final transplenic digital subtraction venography acquired after deployment of two uncovered metal stents (Omnilink 10 × 29 mm and Absolute Pro 9 × 40 mm, Abbott, Abbott Park, IL) to create a porto-portal bypass. Suboptimal liver parenchyma opacification was due to extensive intraprocedural embolism but liver portal flow dramatically improved at colour Doppler ultrasound few days after the procedure

An 8-year-old boy affected by severe portal hypertension resulting from congenital portal cavernoma was referred to our hospital for assessment of Meso-Rex bypass eligibility. Besides splenomegaly and thrombocytopenia (69,000 platelets/L), endoscopy revealed refractory red colour-positive F1 oesophageal varices and the Rex recessus was proved to be viable at wedge hepatic venography. Meso-Rex bypass surgery was attempted via laparotomy in 2022 but was aborted due to the intraoperative detection of moderate liver steatosis, which eventually was not confirmed on wedge hepatic biopsy. Upon multidisciplinary discussion, a percutaneous portal vein recanalization attempt was performed via transplenic venography in 2023 but failed. A new simultaneous attempt via transhepatic and transplenic venography was carried out in 2024 (Fig. 1): the transhepatic access was used to navigate the native intrahepatic portal branches and attempt retrograde recanalization, while the transplenic one for antegrade attempts. Despite the use of guidewires dedicated to revascularization of peripheral arterial chronic total occlusions (PT-Graphix 0.014″ and V18 0.018″, Boston Scientific, Marlborough, MA; Hi Torque Command 0.014″; Abbott, Abbott Park, IL) and low-profile microcatheters (Progreat Lambda 1.9F, Terumo, Tokyo, Japan), anatomical recanalization of the extrahepatic tract of the portal vein failed. Due to the proximity of the transhepatic microcatheter tip with the transplenic one on the posterior side of the hepatic hilum in relation to the preoperative computed tomography (CT) scan, and the anterior visualization of biliary and arterial structures on ultrasound, a non-anatomical revascularization was achieved by means of the “gun-sight” technique, using two snare catheters and a fluoroscopy-guided puncture. Portal vein reconstruction was completed by angioplasty and stenting (Omnilink Elite 10 × 30 mm, Absolute Pro 9 × 40 mm, Abbott, Abbott Park, IL) with restoration of a normal inflow into the native intrahepatic portal system and without periprocedural complications. Bare metal stents were chosen to respect portal vein branching from the hepatic hilum where the most proximal stent landed; the risk of bleeding was anticipated to be minimal, being the gap very short and intrahepatic based on the preoperative CT scan. The child was discharged asymptomatic 1 week after the procedure. Six months after portal vein recanalization, normal portal flow was visible at colour Doppler ultrasound, platelet count improved (131,000 platelets/L) and endoscopy proved disappearance of varices.

## Technique 2

**Fig. 2:**
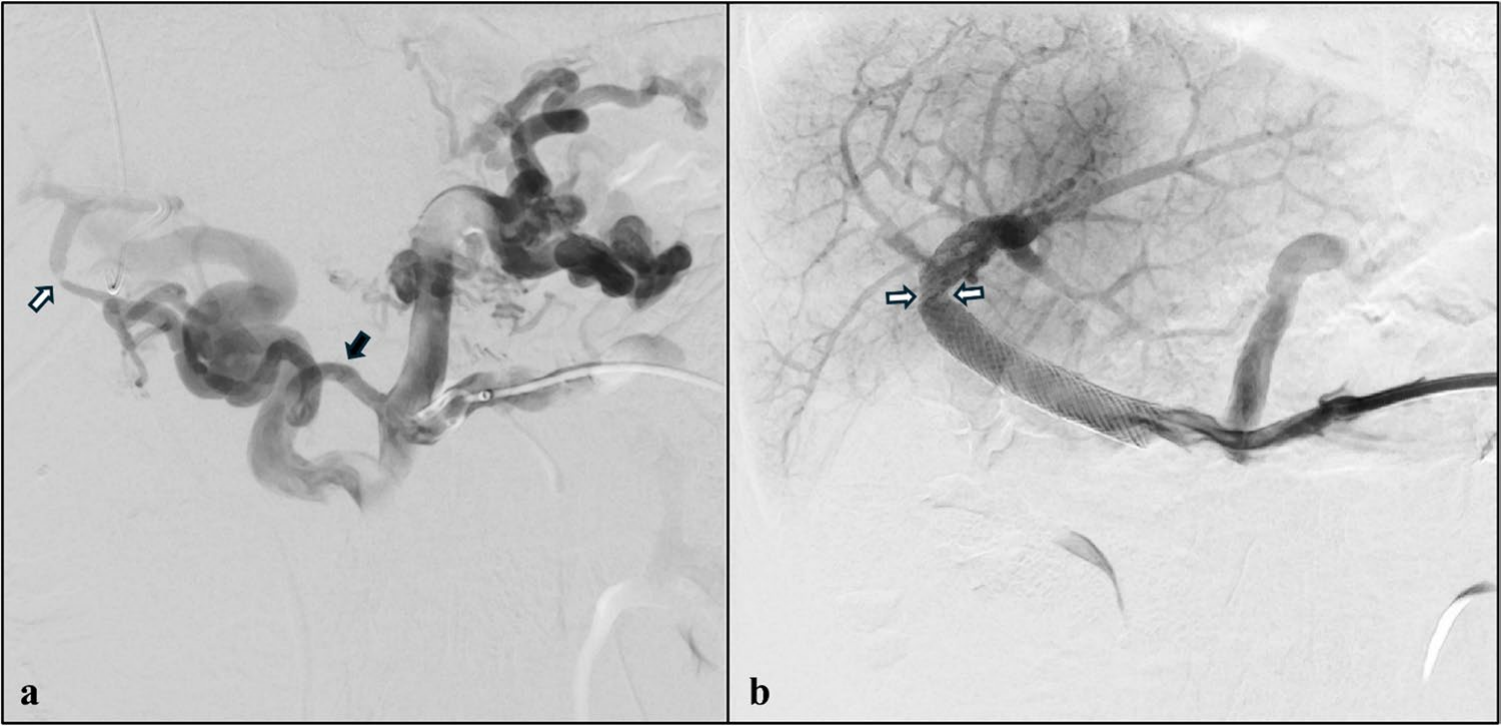
A 14-year-old boy with extrahepatic portal vein obstruction resulting from perinatal thrombosis.** a** Posteroanterior view of the transplenic digital subtraction venography shows cavernous transformation of the portal vein. A small, tortuous vessel (*black arrow*) connects the superior mesenteric vein to the right intrahepatic branch of the portal vein, providing a spontaneous porto-portal bypass, albeit with a focal extrahepatic stenosis (*white arrow*). **b** Posteroanterior view of the final transplenic digital subtraction venography shows portal vein recanalization achieved after angioplasty and stenting (Omnilink 10 × 29 mm, Abbott, Abbott Park, IL; Viabahn VBX 9 × 59 mm, Gore, Newark, DE; Wallstent 12 × 40 mm, Boston Scientific, Marlborough, MA) of the small tortuous vessel. The *white arrows* indicate a tiny concentric notch corresponding to the site of tight stenosis

A 14-year-old boy with known portal cavernoma and severe portal hypertension was referred to the intensive care unit of our centre for acute variceal bleeding and melena (haemoglobin nadir of 7 g/dL). Besides splenomegaly, endoscopy revealed red colour-positive F2-F3 gastric and oesophageal varices that were ligated. The child was screened for Meso-Rex shunt feasibility, and during assessment, a colour Doppler ultrasound scan revealed the patency of tiny native intrahepatic portal branches in the right hepatic lobe. Upon multidisciplinary discussion, a percutaneous portal vein recanalization was attempted via simultaneous transhepatic and transplenic approaches (Fig. 2). The transhepatic venography proved normal opacification of the intrahepatic portal system with viability of the Rex recessus. The transplenic venography showed cavernous transformation of the extrahepatic portal vein but revealed the presence of a spontaneous stenotic porto-portal bypass. The stenosis was easily crossed via the transplenic access, and it was treated with angioplasty and stenting (Omnilink Elite 10 × 30 mm, Abbott, Abbott Park, IL; Viabahn VBX 9 × 39 mm, Gore, Newark, DE; Wallstent 12 × 40 mm, Boston Scientific, Marlborough, MA) with restoration of normal flow into the native intrahepatic portal system. The child presented transient direct hyperbilirubinemia (up to 2.8 mg/dL) which spontaneously resolved in 1 week and was discharged asymptomatic. Six months after portal vein recanalization, normal portal flow was visible at colour Doppler ultrasound, platelet count was within normal ranges and endoscopy proved disappearance of varices.

## Discussion

Following the experience in inoperable paediatric patients, we are accumulating evidence that portal vein recanalization is minimally invasive, it has relatively high technical and clinical success rates even in inoperable patients, and it does not hamper a subsequent surgical approach. Based on these observations, we currently consider a portal vein recanalization attempt before surgery, following a careful multidisciplinary evaluation and an exhaustive information to the patient’s family.

We described two innovative techniques for portal vein recanalization which allowed to restore the physiological portal flow to the liver through a radiologically-created bypass, even when the catheterization of the native thrombosed portal vein failed. Owing to technical improvements, percutaneous recanalization of chronic venous obstructions is gaining popularity. Sharp recanalization techniques have been described for the management of chronic total occlusion of central veins [6], providing exceptional results that must be weighted with the risk of procedure-related complications, as for example pericardial bleeding. Portal vein recanalization is an innovative technique with promising results in different clinical scenarios. If on the one hand this technique is widely adopted and effective to manage portal vein obstruction after liver transplantation, few reports describe its application in patients with native liver affected by non-cirrhotic extrahepatic portal vein obstruction [7], with only preliminary studies in children [5]. In most cases, portal vein recanalization is performed through the anatomic route while it has been reported non-anatomically very exceptionally [8]. The reason why non-anatomical revascularization of the portal vein is challenging is inherent to the density of vital structures encountered at the hepatic hilum (i.e. hepatic artery; common bile duct). The interventional radiologist must be aware of potential complications, including arterial bleeding [5], biliary obstruction and leakage and acute portal vein thrombosis, and he must have technical skills to eventually face such circumstances with appropriate devices like stent grafts and biliary drainages. Moreover, the interventional radiological team must be supported by dedicated paediatric anaesthesiologists and surgeons prepared to manage severe complications. We remark that no complications occurred in our cases of non-anatomical portal vein recanalization and the clinical outcome was favourable for both children with clinical regression of portal hypertension 6 months after treatment.

Cutting-edge devices for chronic total occlusion revascularization, including extra-thin guidewires and coronary angioplasty microcatheters, are useful in both anatomical and non-anatomical portal vein recanalization. If anatomical portal vein recanalization may be achieved with angioplasty alone, non-anatomical portal vein recanalization always needs metal stenting. There is not a favourite device but, as a general rule, balloon-expandable metal stents that can be post-dilated are preferred in younger patients with a significant potential to grow. Technical development now provides metal devices which are highly biocompatible, for example, cobalt-chromium and nickel-titanium alloys and polytetrafluoroethylene (PTFE) coating. The advantages of new metal alloys involve the availability of devices with a very thin strut design which reduces interaction (potentially thrombogenic) with the endothelium, although providing high radial force against recoiling. Mostly in adult study, data about long-term tolerability of these devices have emerged.

A close post-procedural clinical and imaging follow-up is recommended. Anticoagulation therapy is necessary, despite no consensus exists. After a successful portal vein recanalization, we prescribe a therapeutic dose of heparin for 3–6 months to all complex cases judged at high risk of thrombosis. On an individual basis, prophylactic heparin may be coupled with a single antiplatelet agent for the same time-span.

The downsides of the portal vein recanalization technique, and in particular the non-anatomical one, relate to the long procedural times with considerable exposure to ionizing radiations and iodinated contrast agents, with additional procedures often required to manage obstruction recurrence; though, the benefits might outweigh the costs. Interventional radiologists should be aware of these innovative techniques that might improve the management of non-cirrhotic extrahepatic portal vein obstruction in children who are not eligible for standard treatments. Nevertheless, we acknowledge that our approach may not be accepted or applied in all centres, especially those which lack specific expertise, and that large cohorts and long-term follow-up are still lacking. Therefore, we are cautious in generalizing conclusions on the efficacy of the proposed techniques.

## Data Availability

No datasets were generated or analysed during the current study.
